# Genome Sequence of *Fusarium graminearum* Isolate CS3005

**DOI:** 10.1128/genomeA.00227-14

**Published:** 2014-04-17

**Authors:** Donald M. Gardiner, Jiri Stiller, Kemal Kazan

**Affiliations:** Commonwealth Scientific and Industrial Research Organisation (CSIRO) Plant Industry, Queensland Bioscience Precinct, Brisbane, Queensland, Australia

## Abstract

*Fusarium graminearum* is one of the most important fungal pathogens of wheat, barley, and maize worldwide. This announcement reports the genome sequence of a highly virulent Australian isolate of this species to supplement the existing genome of the North American *F. graminearum* isolate Ph1.

## GENOME ANNOUNCEMENT

*Fusarium graminearum* causes *Fusarium* head blight (FHB) of wheat and barley and ear rot of maize (1, 2). The *F. graminearum* genome (isolate Ph1) was one of the first fungal pathogen genomes sequenced (3), and the availability of this genome has been instrumental in facilitating research on this species. The genome sequence of an Australian isolate (CS3005) of *F. graminearum sensu stricto* is reported here. CS3005 was isolated from barley showing FHB symptoms near Warwick, Queensland, Australia, in November 2001 (4). CS3005 is virulent toward wheat, barley, and maize and has been used extensively in our laboratory (5–10).

Fungal DNA for sequencing was extracted from freeze-dried mycelia using a QIAgen DNeasy plant DNA minikit. An indexed Illumina TruSeq library was prepared by the Australian Genome Research Facility, Melbourne, Australia, and sequenced using 100-bp paired-end reads on an Illumina HiSeq 2000 instrument using approximately 1/12 of a sequencing lane. A total of 2.12 Gbp of raw data were generated from this sequence run. The reads were imported into the CLC bio Genomics Workbench version 6.5.1 and quality trimmed (quality limit, 0.05, with no more than two ambiguous residues and two 5′ nucleotides removed). Mitochondrial reads were removed by mapping all reads to the Ph1 mitochondrial genome downloaded from the Broad Institute’s *Fusarium* database (http://www.broadinstitute.org/annotation/genome/fusarium_group/MultiHome.html). The assembly of the remaining genomic reads to the Ph1 reference genome suggested that only 97% of the Ph1 genome was shared with CS3005 and approximately 1 Mb of sequence was unique to each isolate. These analyses indicated a *de novo* assembly, and *ab initio* gene predictions were required for the CS3005 genome. Genomic reads were *de novo* assembled in CLC bio Genomics Workbench using default parameters, with the scaffolding option selected. A total of 36.6 Mbp of sequence was assembled into 424 contigs at an average sequence depth of 40-fold. The assembly L_50_ (minimum number of contigs for which 50% of the assembly is contained) is 27 contigs, with an *N*_50_ length of 460 kbp.

To annotate the protein-coding genes of CS3005, Augustus version 3.0.1 (11) was used to *ab initio* predict genes with guidance from the Ph1 coding sequences provided to Augustus, following BLAT (version 35x1) (12) alignment (85% identity cutoff, with only unique hits retained) to the CS3005 contigs. Regions in which Augustus predicted genes were masked using the maskFastaFromBed script in BEDTools version 2.14.3-1 (13). To supplement the Augustus gene predictions, the masked genome sequence was then used as input into Fgenesh run in the MolQuest 2.4.3 package. The Augustus and Fgenesh predictions were then combined. BLAST reciprocal best hit analyses with ≥80% identity were performed to identify CS3005 homologues of the Ph1 gene sets (downloaded from the Broad website). Unique gene identifiers from 12,176 Ph1 genes were transferred to CS3005 to indicate orthologous genes. For example, *TRI5* has the locus tags FGSG_03537 and FG05_03537 in Ph1 and CS3005, respectively. A total of 1,179 CS3005 genes from the combined Augustus and Fgenesh predictions did not have reciprocal best BLAST hits to Ph1 and were given locus tags with numbers starting from 30001 in CS3005. The total number of predicted protein-coding genes in the CS3005 genome is 13,355.

### Nucleotide sequence accession numbers.

This whole-genome shotgun project has been deposited in DDBJ/ENA/GenBank under the accession no. JATU00000000. The version described in this paper is the first version, JATU01000000.
